# Characterization and genome analysis of virulent phage against phytopathogen *Pantoea ananatis* and its potential biocontrol application

**DOI:** 10.1186/s12866-026-04891-2

**Published:** 2026-03-25

**Authors:** Huda Husien Badr, Kamel M. Elhalag, Abdelmonim Ali Ahmad, Mohamed A. Nasr-Eldin

**Affiliations:** 1 Bacterial Diseases Research Department, Plant Pathology Research Institute, Agricultural Research Center (ARC), Giza, Egypt; 2 Department of Plant Pathology, Faculty of Agriculture, Minia University, El-Minia, 61519 Egypt; 3 Botany and Microbiology Department, Faculty of Science, Benha University, Benha, 13511 Egypt

**Keywords:** Lytic Phage, Genome analysis, *Pantoea ananatis*, Biological control

## Abstract

**Background:**

*Pantoea ananatis* is an emerging phytopathogen that causes leaf blight and fruit or bulb rot diseases in a wide range of economically important plant species, resulting in severe yield loss. This study investigates the isolation and characterization of a virulent bacteriophage for potential biocontrol of bacterial rot and leaf blight diseases caused by *P. ananatis*.

**Results:**

Virulent phage was isolated from surface agricultural drainage water collected in Dakahlia Governorate, Egypt using a single plaque purification method. Morphological analysis classified phage PanM1EGY as Myovirus-like. The optimal multiplicity of infection (MOI) was 0.1; one step growth curve revealed a latency period of 30 min and a burst size of approximately 252 ± 2 PFU/ cell. Environmental stability tests showed that phage PanM1EGY maintained activity across a broader temperature range (4 °C–70 °C) and under pH conditions ranging from 5 to 11. Results of the UV and NaCl tolerance experiments showed that the phage titer did not change following 5 min of UV exposure or incubation at a 5% NaCl concentration. In vitro assay indicated that the phage effectively reduced and prevented *P. ananatis* growth. The genome of PanM1EGY consisted of a double-stranded DNA with a total length of 77,516 base pairs and a GC content of 37%. A total of 131 coding sequences were predicted. *In planta* experiments utilizing phage treatments demonstrated that treated potato tubers, onion bulbs, and garlic cloves exhibited inhibition of rot disease symptoms compared to untreated controls. Likewise, the phage treatment effectively prevented the development of leaf blight in strawberry leaves.

**Conclusion:**

These results highlight the possibility of using *P. ananatis*-infecting phage as a sustainable biocontrol method to manage the symptoms of the disease induced by rot and leaf blight across variety of plants.

**Supplementary Information:**

The online version contains supplementary material available at 10.1186/s12866-026-04891-2.

## Background

*Pantoea ananatis* is a Gram-negative, facultative anaerobic bacterium within the phytopathogenic Enterobacteriaceae family. It has emerged as a significant pathogen affecting numerous economically vital crops such as maize, rice, wheat, pineapple, strawberry, soybean, cotton, and onion [1–3]. The bacterium is known to cause a wide array of disease symptoms, including leaf blight, streaks, spots, stem and fruit rot, top node necrosis, and grain discoloration, resulting in major yield reductions globally [4–7].

Initially recognized as a plant epiphyte and endophyte with plant growth-promoting attributes, *P. ananatis* has increasingly been implicated in plant diseases across a broad spectrum of crops. This dual role as both a potential symbiont and pathogen has raised concerns in agricultural and scientific communities [8]. Its pathogenic activity has been documented in diverse agro-ecological zones, impacting crops such as rice (*Oryza sativa*), maize (*Zea mays*), wheat (*Triticum aestivum*), strawberry (*Fragaria* × *ananassa*), and cucumber (*Cucumis sativus*) across countries including China, the USA, Egypt, India, Russia, Poland, Brazil, and Malaysia [3].

Recent studies have highlighted the expanding distribution and increasing occurrence of *P. ananatis* in agriculture. In rice cultivation, the bacterium has been linked to leaf blight and grain discoloration in regions such as Kerala, India [9], Guangdong, China [10], Russia [11], and Brazil [12]. In maize, it is recognized as the causative agent of white spot disease in both China [6] and the United States (USA) [13]. Similarly, wheat fields in the USA [4, 14], China [15], and Poland [16] have experienced outbreaks of leaf streak and blight due to *Pantoea* spp. Strawberry and cucumber plants have also been affected by bacterial blight, crown necrosis, and stem rot in Egypt and China [3, 17, 18].

The widespread emergence and impact of *P. ananatis* underscore the urgent need for innovative and sustainable disease management strategies. The rise of disease resistance and environmental considerations poses problems for traditional chemical control methods. As a result, biological control strategies, especially those utilizing actinobacteria and other beneficial microbes, have gained attention due to their promising antagonistic activity against *P. ananatis* and *Pantoea* spp. [19–21].

In Egypt, *Pantoea spp.* has become an increasingly important pathogen, causing leaf blight and fruit rot in key crops such as strawberry, bean, tomato and onion. This has led to significant agricultural losses, with estimated yield declines ranging from 15 to 25%, threatening grower livelihoods and national food security [3, 21–23].

In recent years, bacteriophage (phage) therapy has emerged as a promising, environmentally friendly alternative for controlling bacterial plant pathogens. Phages are viruses that specifically infect bacteria, offering a highly targeted biocontrol approach that minimizes harm to non-target microbes and the surrounding ecosystem [20, 21, 24]. Their host specificity and ability to replicate within bacterial cells make phages an attractive component of integrated pest management programs [21, 24]. Although phage-based treatments have shown potential against several phytopathogens, including some *Pantoea* species [25, 26], there remains a need for further research to improve their application under field conditions and across different crop systems.

To date, 22 Pantoea-infecting bacteriophages have been sequenced and made publicly accessible [21, 24–27]. These phages span all three major tailed phage morphotypes, myoviruses, siphoviruses, and podoviruses, with genome sizes ranging from 36,790 to 149,913 base pairs and GC content between 39% and 55.35% [27].

The main objective of this study is to isolate and characterize a novel *P. ananatis* bacteriophage physically, biologically, and genomically, and to test the hypothesis that it is a stable, safe, and highly effective biocontrol agent capable of significantly reducing *P. ananatis*-induced diseases *in planta* across different vegetable crops.

This study focuses on the isolation and identification of a novel bacteriophage targeting *P. ananatis*, along with a detailed characterization of its physical, biological, and genomic features. Additionally, the phage’s potential as a biocontrol agent will be assessed through *in planta* experiments on various vegetable crops. This work represents the first comprehensive report that includes isolation, functional analysis, genome characterization, and *in planta*-relevant evaluation of a *P. ananatis*-infecting bacteriophage. The goal is to establish this phage as a viable and sustainable tool for managing diseases caused by *P. ananatis*.

## Methods

### Bacterial strains and culture conditions

The bacterial strains used in this study, including *Pantoea ananatis*, were maintained in the culture collection of the Bacterial Diseases Research Department at the Plant Pathology Research Institute, Agricultural Research Center, Giza, Egypt. Preservation was carried out using 25% (v/v) glycerol at a temperature of − 30 °C. In total, five strains of *P. ananatis* and 22 other bacterial species were examined to assess the phage host range and specificity, as outlined in Table 1. Among them, the *P. ananatis* strain Badr HH1, which was biochemically, molecularly identified and was deposited under the accession number PV362860, served as the host for phage propagation and as the indicator organism for *in-planta* phage application. Bacterial and phage cultures were maintained using King’s broth and nutrient agar (NA) media. *P. ananatis* produces yellow, shiny, and smooth colonies on both media, the colonies are 3- 4 mm in diameter, circular, and have a drop-shaped appearance. *P. ananatis* strain Badr HH1was Catalase positive, Oxidase negative, positive for indole production.

**Table 1:** Bioactivity and specificity of phage PanM1EGY against a panel of pathogenic and antagonistic bacteria

Bacterium		Bacterial species	Strain^a^	Spot test*	Plaque assay*
	*Pantoea* spp.	*Pantoea ananatis*	Badr HH1	+	+
		*Pantoea ananatis*	PaH1	+	+
		*Pantoea ananatis*	KHM	+	+
		*Pantoea ananatis*	PaA	+	+
		*Pantoea ananatis*	PaH2	+	+
Pathogenic bacteria	*Pectobacterium* spp*.*	*Pectobacterium brasiliense*	KMBR1	-	-
			PbrH1	-	-
		*Pectobacterium* *Atrosepticum*	MH3c	-	-
		*Pectobacterium carotovorum*	100H	-	-
		*Pectobacterium aroidearum*	P.ash	-	-
	*Pseudomonas* spp.	*Pseudomonas marginalis*	PMH2	-	-
	*Dickeya* spp.	*Dickeya chrysanthemi*	300R1	-	-
	*Lysinibacillus boronitolerans*		LBH4	-	-
	*Siccibacter turicensis*		STH5	-	-
	*Corynebacterium* spp.	*Corynebacterium glutamicum*	CgH3		
	*Robbsia andropogonis* (*Burkholderia andropogonis*)		Ba	-	-
	*Ralstonia solanacearum* (Phylotype IIA sequevar 1)		K6	-	-
			K9	-	-
			K10	-	-
	*Rhizobium radiobacter* (*Agrobacterium tumefaciens*)		AGt	-	-
	*Erwinia amylovora*		Ea	-	-
Antagonistic bacteria	*Pseudomonas japonica*		447	-	-
	*Pseudomonas putida*		600B	-	-
	*Pseudomonas fluorescense*		AH	-	-
	*Enterobacter aerogenes*		114	-	-
	*Bacillus thuringiensis*		400B	-	-
	*Bacillus subtilis*		500B	-	-
	*Serratia marcescens*		100B	-	-
	*Pseudomonas aeruginosa*		177	-	-
	*Stenotrophomonas maltophilia*		300B	-	-

^a^All bacterial strains used in this study were isolated in Egypt [28–31] and maintained at the culture collection of the Bacterial Diseases Research Department, Plant Pathology Research Institute, Giza, Egypt.

The results were recorded as follows: (+), spot titration and plaques positive; (-), spot titration and plaques negative

### Isolation, purification and propagation of bacteriophage

Phage were isolated from surface agricultural drainage water collected from various sites in Aga, located in the Dakahlia Governorate, Egypt, during the summer of 2024 according to method described by Abou Zeid et al. [32]. Initially, 100 ml water samples were subjected to centrifugation at 6,000 × g for 5 min at 25 °C to eliminate solid debris and naturally occurring bacteria. Subsequently, 10 mL of the clarified drainage water was mixed with 20 mL of *P. ananatis* strain Badr HH1 (OD_600_ = 0.5) in 100 mL of sterile King’s broth then incubated at 28 °C for the entire night. Following incubation, bacterial cells were removed from the mixture by centrifuging and filtering it through 0.22 μm membranes, producing a crude phage lysate. This lysate was then used in a double-layer agar plaque assay to isolate individual phages. A single plaque was selected and transferred to a fresh culture containing the host strain for overnight propagation. The culture was then centrifuged at 5,000 × g for 10 min and passed through a 0.22 μm filter to further purify the phage. This purification process was repeated at least three times to ensure the phage’s purity.

The propagation of the phage was done over three different frequently phases. In the first phase, a purified plaque was cultured with 30 µL of host bacteria in 3 mL of King’s liquid medium, incubated for 8 h at 28 °C with shaking at 150 rpm. Afterward, the culture was centrifuged (5,000 × g for 10 min) and filtered through a 0.22 μm syringe filter.

In the second phase, the enriched phage preparation was scaled up by co-culturing with 1 mL of *P. ananatis* Badr HH1 in 100 mL King’s broth under the same conditions. The second phase, includes the use of the phage lysate from the first propagation stage (start-up inoculum) to inoculate 100 ml of fresh bacterial suspension, incubated for 24 h, centrifuged, and filtered. The phage titre was also calculated, which estimated to be 10^10^ PFU/mL.

The third phase (high-titer phage production): included the use of the phage lysate from the second phase (initial high-titer lysate, typically 10^10^ PFU/mL), to inoculate 1500 ml of fresh bacterial suspension at an optimal multiplicity of infection (MOI). Where, a total of 1.5 L of bacterial culture was grown. When the optical density at 600 nm (OD_600_) of the *P. ananatis* strain Badr HH1 reached 0.5, the phage lysate with an initial high-titer (10^10^ PFU/mL) was added at an optimal MOI. After further growth for 24 h, the bacterial cells were removed by centrifugation at 5,000 × g for 10 min at 4 °C. The supernatant was passed through a 0.22-µm pore-size membrane filter, and the lysate had the highest phage titer reaching 4.59 × 10^14^ PFU/mL. Phage particles were precipitated by centrifugation at 15,000 × g for 2 h at 4 °C and then suspended in SM buffer. Purified phages were stored at 4 °C until use.

### Determination of host range and efficiency of plating

The host range of the phage was evaluated using a spot assay based on the double-layer agar method, as outlined by Kropinski et al. [33]. To perform the test, 200 µL of *P. ananatis* strain Badr HH1 in the mid-logarithmic growth phase was mixed with 3 mL of molten soft agar (0.4% w/v) and uniformly poured over a base layer of King’s agar medium (1.5% w/v agar). After the overlay solidified, 5 μL of the phage suspension was carefully applied onto the surface. Once the drops had dried, the plates were inverted and incubated at 28 °C for 12 h. The appearance of clear lysis zones indicated bacterial strains susceptible to phage infection.

Furthermore, the efficiency of plating (EOP) was determined using a plaque assay protocol previously described by Kutter et al. [34]. Each assay was conducted in three independent biological replicates to ensure reliability and reproducibility.

### Determination of propagated-bacteriophage morphology

Phage morphology was examined following the procedure outlined by Sasaki et al. [35]. Concentrated phage suspensions (10^14^ PFU/mL) were prepared for visualization using transmission electron microscopy (TEM) with negative staining. Specifically, 10 μL of the purified phage solution was applied to a copper grid coated with a carbon film and left to stand for 15 min to allow particle adsorption. The grid was then gently wiped with filter paper, stained for two minutes with 2% uranyl acetate (pH 4.0), and then blotted once more to get rid of any remaining stain. Following air drying, the samples were examined using a transmission electron microscope (JEOL JEM-2100, Japan Electron Optics Laboratory Co., Ltd) at the Nano Tech Company in Egypt. To determine phage dimensions, the head diameter and tail length were measured using at least five individual phage particles, that have the ability to inhibit bacterial growth.

### Determination of optimal multiplicity of infection (MOI)

Logarithmic phase cultures of *P. ananatis* strain Badr HH1 were combined with purified phage suspensions at various multiplicities of infection (MOIs), ranging from 10^3^ to 10⁻^3^. The mixtures were incubated with shaking at 28 °C and 150 rpm for 8 h. Following incubation, the samples were filtered using 0.22 μm syringe filters. The resulting filtrates were serially diluted and used to prepare double-layer agar plates. After incubation, plaques were counted, and the average count was used to calculate phage titers. The MOI that yielded the highest titer of plaque-forming units (PFU) was considered the optimal MOI. This experiment was conducted in triplicate to ensure consistency and accuracy.

### Determination of one step growth curve

A one-step growth curve test was performed to ascertain the latent period and burst size of the isolated phage [36]. Briefly, 5 mL of an exponential-phase culture of *P. ananatis* strain Badr HH1 was centrifuged for 5 min at 4 °C at 8,000 × g. To get an optical density (OD_600_) of 0.4 to 0.5, the resultant pellet was subcultured in 10 mL of King's broth. After that, sterile King's broth medium was used to get the bacterial concentration down to about 10^9^ CFU/mL [36]. The phage was introduced to the bacterial suspension at a concentration of 10⁸ PFU/mL in order to achieve a multiplicity of infection (MOI) of 0.1. For 15 min, the mixture was let to stand at room temperature in order to promote phage adsorption.

Subsequently, the mixture was centrifuged at 8,000 × g for 10 min to eliminate un-adsorbed phage particles. The pellet was resuspended in 100 μL of King’s broth and then transferred into 9.9 mL of fresh King’s broth. This culture was incubated at 28 °C in a shaking incubator at 150 rpm for 1.5 h. At 10-min intervals over a 90-min period, 200 μL samples were collected. To determine the phage titer, each sample was subjected to the plaque assay [34]. By dividing the final phage titer by the original number of infected bacterial cells during the latent phase, the burst size was determined following the approach of Sun et al. [37]. All experiments were performed in triplicate to ensure reproducibility.

### Determination of biological characteristics of phage

#### Temperature and pH stability assay

The biological properties of phage were investigated using methods described by Huang et al. [38] with some slight adjustments. The phage stocks solution (10^14^ PFU/ml) were incubated at 4 °C, 28 °C, 40 °C, 50 °C, 60 °C, 70 °C, and 80 °C for 1 h in order to assess the titers using the double-plate agar method for temperature stability. To ensure pH stability, phage solutions (10^14^ PFU/mL) were combined with various SM buffers (pH 3–11, adjusted with Hydrochloric acid (HCl) or Sodium Hydroxide (NaOH) at 28 °C. Samples were collected at 1 h to calculate the titers using the double-plate agar method. All tests were repeated three times.

#### UV irradiation and salinity tolerance assay

In order to test for UV stability, 2 mL of phage samples containing a titer of 10^14^ PFU/mL were exposed to UV radiation (λ = 365 nm; 320 mW/m2) for 5, 10, 15, and 20 min, respectively [39]. The samples were collected to determine the titer.

Phage samples were examined for salinity stability at various NaCl concentrations, ranging from 5 to 20%. This was done by adding phage aliquots to various amounts of NaCl solutions and letting them sit at room temperature for 1 h. The plaque assay was used to count the phages following the incubation. All tests were repeated three times.

### Phage genome sequencing and bioinformatics analysis

Standard molecular biological techniques for DNA isolation, digestion with restriction enzymes and other nucleases were followed as described by Sambrook and Russell [40]. Phage DNA was extracted from a high titer purified phage particles solution (minimum of 10^8^ PFU/mL) using using the phenol extraction method as described by Sambrook and Russell [40]. DNA purity and concentration were determined using the Nanodrop ND-1000 spectrophotometer (Thermo Fisher Scientific, Wilmington, USA) based on Karumidze et al. [41]. De novo paired-end sequencing was performed commercially by commercially by SeqMatic (Fremont, CA, United States) on an Illumina MiSeq with 2 × 300 bp reads and the genome sequence was assembled using Spades v3.12 by AmpSeq (Gaithersburg, Maryland, USA). Open reading frames (ORFs) exceeding 30 amino acids (aa) were identified using method described by Arndt et al. [42], GeneMarkS [43]. To assign possible functions for each predicted ORF, homology searches using BLASTN and BLASTP and PSI-BLAST [44]. An e-value threshold of e − 4 or lower was established to qualify two proteins as a match. Complete genome sequence of the phage was analyzed against the GenBank database via BLASTn in order to identify closely related phages, and the Average Nucleotide Identity (ANI) value was calculated using the OrthoANI tool (version 0.93.1) via the EzBioCloud online platform (https://www.ezbiocloud.net/tools/ani) to assess the overall similarity between genome sequences [45].

#### Determination of lytic activity of the phage

The *P. ananatis* in the logarithmic phase (500 μL) and the phage (500 μL) at different MOIs (0.001, 0.01, 0.1, 1, 10, 100, and 1000) were mixed and added into 39 mL of King’s broth medium. The samples were incubated at 28◦C for 12 h, and the Optical Density at 600 nm (OD_600_) was measured at 0, 1, 2, 3, 4, 5, 6, 7, 8, 9, 10, 11 and 12 h, respectively [46]. Three biological replicates were performed for each experiment.

#### In planta assessment for the phage suppressive effect against *P. ananatis* on potato, onion, garlic and strawberry

All vegetable samples used in this experiment were procured from local markets. To evaluate the antibacterial efficacy of the phage against soft rot disease, potato tubers, onion bulbs, and garlic cloves were first surface sterilized using 70% ethanol, rinsed with sterile distilled water, and then air-dried under a laminar flow hood. A single well was created in each vegetable sample, into which 50 μL of *P. ananatis* suspension (10^9^ CFU/mL) was introduced, followed by 50 μL of the phage solution at a multiplicity of infection (MOI) of 10^3^.

For post-inoculation handling, potato tubers were sealed using the original cut-out piece and paraffin wax as described by [47]. Onion bulbs were similarly sealed with their respective cut sections, while garlic cloves were covered with sterile, water-moistened filter paper, wrapped in plastic, and maintained at room temperature. Control groups included samples treated only with *P. ananatis* (designated as pathogen controls) and those inoculated with sterile King’s broth (SKB) alone (negative controls). Using the detached leaf approach, biocontrol of *P. ananatis'* using phage PanM1EGY was assessed. The healthy strawberry leaves were surface sterilized for five minutes using a 2.5% sodium hypochlorite solution and rinsed three times with sterile distilled water, allowed to air dry. The bacterial inoculum density was adjusted to 10^9^ CFU/ml. The detached leaves were infiltrated with bacterial suspension followed by the phage solution at a multiplicity of infection (MOI) of 10^3^, put in moisture plates and parafilm-sealed then were placed in a growth chamber. The strawberry leaves were infused with sterile King’s broth (SKB) in the negative control and *P. ananatis* suspension in the positive control. At 28 ± 2 °C, all treated leaves were incubated until symptoms showed up [48].

All treated vegetables were incubated at 28 ± 2 °C, and observed daily for signs of soft rot development. After three days, the diameter of decayed tissue surrounding each well was measured. Tissue maceration was monitored over two-weeks period to assess disease progression. The in-*planta* experiments repeated twice with three replicates for each treatment.

### Statistical analysis

The stability of the phage under varying thermal and pH conditions, together with its performance in response to different NaCl concentrations and UV irradiation, as well as the in vitro growth of *P. ananatis* strains measured 12 h after exposure to phage PanM1EGY at multiplicities of infection (MOIs) of 0.01, 0.1, and 1.0, were subjected to statistical assessment through a one-way analysis of variance (ANOVA). The analysis was conducted using a freely available online platform (http://vassarstats.net/anova1u.html). Before performing ANOVA, the data were tested for normality using the Shapiro–Wilk test and for homogeneity of variances using Levene’s test. To enable post-hoc comparisons among group means, Tukey’s Honest Significant Difference (HSD) test, integrated within the software, was applied.

Furthermore, the impact of phage PanM1EGY on disease severity was evaluated using independent t-tests in Microsoft Excel, comparing the mean values of untreated versus phage-treated *P. ananatis* strains. Statistical significance was established at a threshold of p < 0.05.

## Results

### Isolation *P. ananatis* infecting bacteriophage

**Fig. 1:**
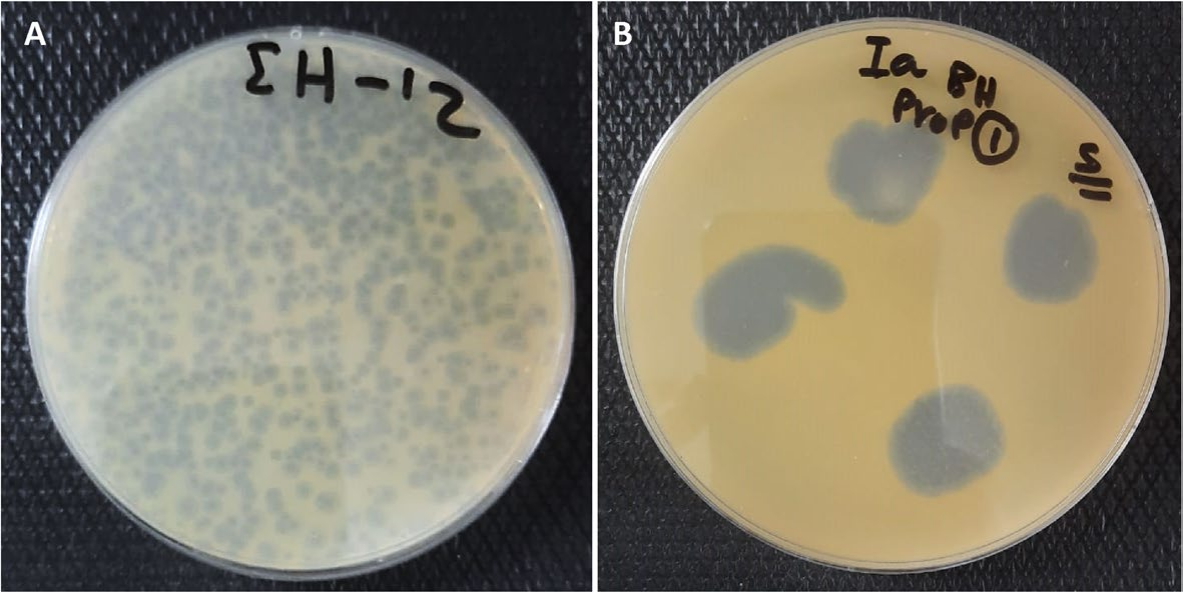
**A** Plaques and (**B**) spot test of the isolated *P. ananatis* infecting phage PanM1EGY, on adouble layered King’s B medium plate using *Pantoea ananatis* as a bacterial host

A bacteriophage was successfully isolated from surface water samples collected in Dakahlia Governorate, Egypt, during the summer of 2024. When incubated overnight at 28 °C on a double-layered King’s plate with a top layer containing 0.75% agar, using *P. ananatis* strain Badr HH1 as the host, the phage formed medium-sized, clear plaques approximately 2–3 mm in diameter (Fig. 1A). Additionally, a clear lysis zone was observed in the spot test, and the phage was designated as PanM1EGY (Fig. 1B).

### Morphological characterization of isolated phages

**Fig. 2:**
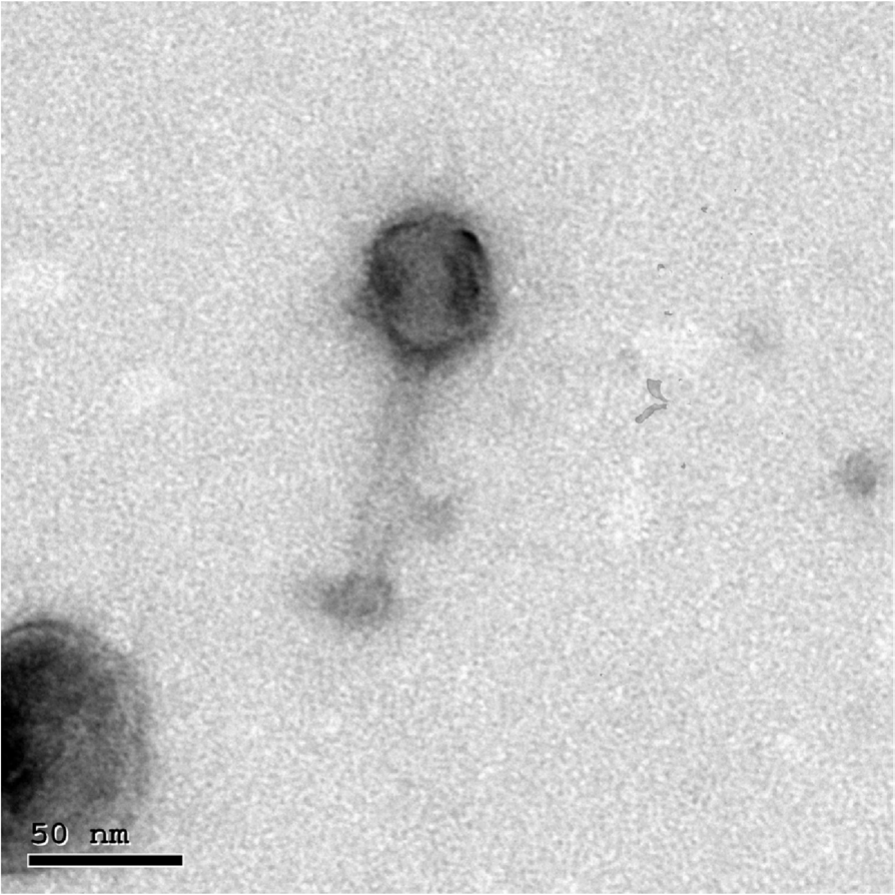
Transmission electron micrographs displaying the morphology of negatively stained PanM1EGY bacteriophage

The morphology was investigated, and the phage was classified with the use of transmission electron microscopy (TEM) (Fig. 2). The phage PanM1EGY exhibited a polygonal head with average capsid dimensions of 52.5 ± 2 × 39.5 ± 1.5 nm (n = 5), along with a long tail measuring approximately 88 ± 1.5 nm in length and 18 ± 3 nm in width (n = 5). It also featured a baseplate and tail fibers with an estimated length of 11.5 ± 2 nm (n = 5). Based on these structural characteristics, the phage morphology is consistent with viruses classified under the class Caudoviricetes, according to the taxonomy guidelines set by the International Committee on Taxonomy of Viruses (ICTV).

### Biological characteristics

#### Host range

Results from spot titration and plaque assays demonstrated that phage PanM1EGY exhibited lytic activity specifically against five *P. ananatis* strains. In contrast, no lytic activity was observed against five *Pectobacterium* species, *Pseudomonas marginalis*, *Dickeya chrysanthemi*, *Lysinibacillus boronitolerans*, *Siccibacter turicensis*, *Corynebacterium glutamicum*, *Burkholderia andropogonis*, *Ralstonia solanacearum* (Phylotype IIA, sequevar 1), *Agrobacterium tumefaciens*, *Erwinia amylovora*, or other nine tested strains of antagonistic bacteria (Table 1).

#### Optimal MOI and one step growth curve

**Fig. 3:**
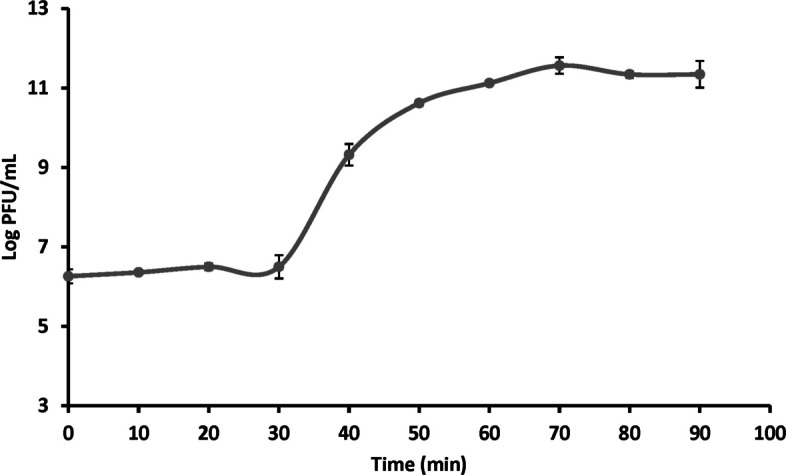
One-step growth curve of phage PanM1EGY

The optimal multiplicity of infection (MOI) for phage PanM1EGY was identified as 10⁻^1^, yielding the highest phage progeny titer of 4.59 × 10^14^ PFU/mL. To investigate the phage’s replication cycle and infection dynamics, a one-step growth experiment was conducted using *P. ananatis* strain Badr HH1 at the optimal MOI of 10^–1^ (Fig. 3). As shown in Fig. 3, the phage exhibited a latent phase lasting approximately 30 min, followed by a rapid replication phase between 30 and 70 min. The burst size was estimated to be around 252 ± 2 PFU per infected cell at 70 min.

#### pH and temperature stability of phage

**Fig. 4:**
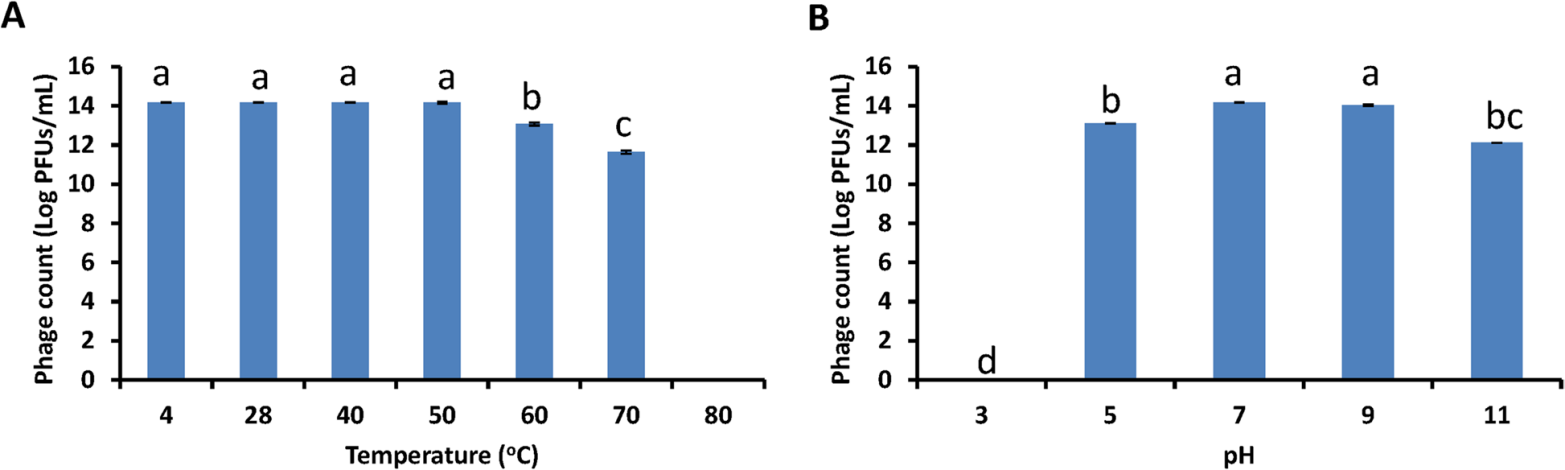
Stability phage PanM1EGY under different temperature (**A**) and different pH (**B**). Different letters indicate statistically significant differences among treatments, while the same letters mean no significant differences

For phage PanM1EGY to be considered a viable antibacterial agent, it must demonstrate resilience under a variety of environmental conditions, including heat, pH extremes, and UV exposure. Temperature stability assays showed that the phage remained stable at 4 °C, 28 °C, 40 °C, and 50 °C with no significant change in titer (Fig. 4A). However, incubation at 60 °C for 1 h led to a titer reduction of 1.09 log units. A more pronounced decline of 2.53 log units was observed after 1 h at 70 °C, and complete inactivation occurred at 80 °C (p < 0.05), indicating that the phage is thermally stable only below 50 °C.

In terms of pH tolerance, phage PanM1EGY maintained stability between pH 7 and 9 (Fig. 4B). At pH 5, the phage titer declined by 1.06 log units after 1 h of exposure, while at pH 11, the reduction was more significant, reaching 2.06 log units. These findings suggest that PanM1EGY is moderately pH-tolerant, especially within the neutral to slightly alkaline range. Given that Egyptian soils typically range from pH 7.7 to 8.5—approximately 7.9–8.5 in the northern delta and 7.7–8.0 in middle and Upper Egypt—the phage appears well-suited for application in local soil environments.

#### UV and salinity stability

**Fig. 5:**
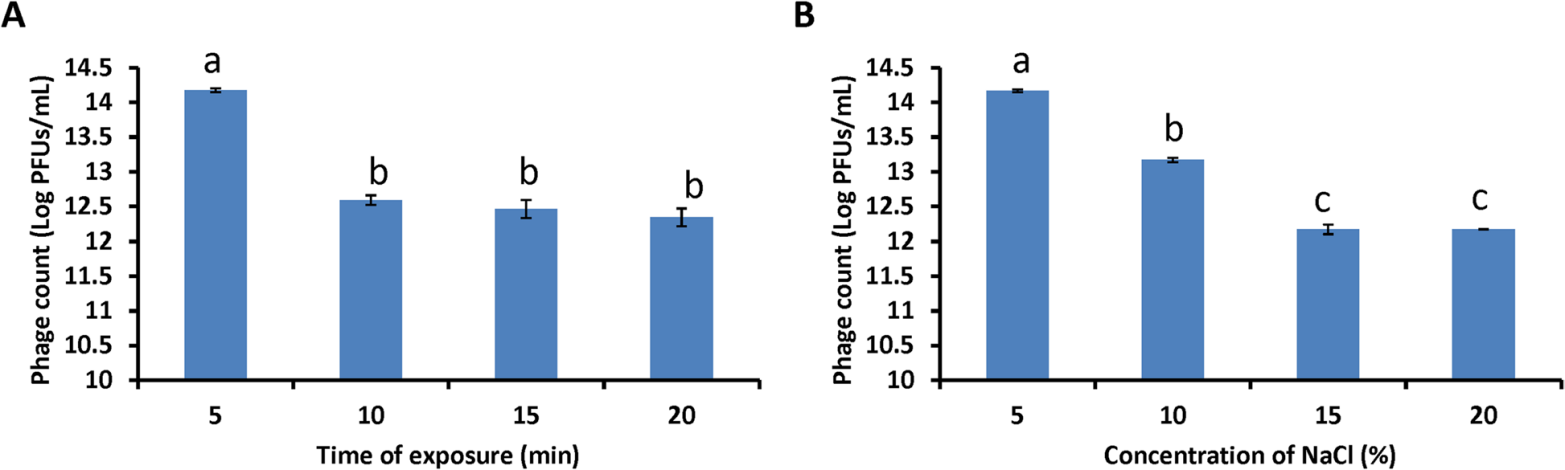
Stability of phage PanM1EGY under and UV exposure (**A**), and different NaCl exposure (**B**). Different letters indicate statistically significant differences among treatments, while the same letters mean no significant differences

UV irradiation experiments showed that the titer of phage PanM1EGY declined upon exposure to ultraviolet light (Fig. 5A). After 10 min of UV exposure, the phage titer dropped by approximately 1.52 log units, reaching 12.59 log. Prolonging the exposure to 20 min further reduced the titer to 12.34 log units.

Similarly, NaCl tolerance assays indicated a decrease in phage viability under high-salt conditions (Fig. 5B). After 1 h of incubation in 10% NaCl, the phage titer declined by about 1.00 log unit to 13.17. When the salt concentration was increased to 20%, the titer was further reduced to 12.17 log units.

### Phage genome sequencing and bioinformatics analysis

#### Genome accession number of phage PanM1EGY

The complete genome sequence of *Pantoea ananatis* phage PanM1EGY was deposited into GenBank under the accession number PX286759.

#### General genomic features of PanM1EGY phage

**Fig. 6:**
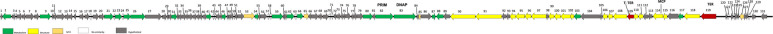
Genomic organization of phage PanM1EGY. Colored arrows indicate the directions and categories of the genes. The color codes are: green, metabolism; yellow, structure; light yellow, lysis proteins; red: terminizes proteins; grey: hypothetical protein. Numbers are annotated ORFs. PRIM, Primase protein; DNAP, DNA polymerase protein;; T-TER, tail terminator;; MCP, phage capsid; TER,terminase

Based on morphological characterization this PanM1EGY belongs to mycoviruses with icosahedral head and long contractile tail (described above, Fig. 6). To further analyze the isolated PanM1EGY phage, genomic DNA was extracted, and genome sequencing performed. PanM1EGYgenome is linear double stranded DNA, degraded by DNase I, but not by RNase A. The phage genome consists of 77,516 bp with a G + C content of 37%, and the genome was scanned for open reading frames (ORFs) of 100 bp or longer using computational software complemented with visual inspection of the genome sequence for ORFs. The search resulted in 131 potential ORFs identified in phage PanM1EGYgenome (Supplementary Table S1, Fig. 6). Sequence analysis using the Orthologous Average Nucleotide Identity (OrthoANI) revealed that isolated phages shared high similarity to other Related phages. Those includes 75.57% with *Pantoea* phage AH01 (accession number “acc: no”: MZ501269.1), 72.16% with *Klebsiella* phage vB_KpnM_BIS47 (acc: no, NC_048656.1), 63.46% with *Pectobacterium* phage POP12 (acc: no, MT560058.1), 62.12% with *Klebsiella* phage 05F01 (acc: no, LC483177), 61.57% with *Erwinia* phage pEp_SNUABM_01*,* (acc: no, NC_048807.1), 61.49% with *Enterobacteria* phage phi92 (acc: no, NC_023693.1), 61.15% with *Pseudaeromonas* phage vB_PpeM_ KLEP7 (acc: no, OM654377.1), 59.87% with Klebsiella phage FKP3 (acc: no, PP895363.1), 59.55% with *Erwinia* phage IT22 (acc: no, OP586623.1), and 58.10% with Pseudomonas phage Astolliot (acc: no, OM982621.1) (Table S1). The majority of identified ORFs found to have roles predicted to be involved in metabolic, virion structure, assembly, DNA packaging, and show a high level of amino acid sequence conservation among other closely related phages (Table S1, Fig. 6). The 131 deduced proteins included (i) 43 proteins that had database homologs of known function, including phage structural proteins, DNA packaging proteins, and proteins involved in DNA replication, transcription, and cell lysis; (ii) 74 hypothetical proteins in the databases and (iii)14 proteins with no similarities in the databases (see Fig. 6, and Table S1 in the supplemental material).

#### Gene Organization of Phage PanM1EGY

The annotated ORFs of phage PanM1EGY can be classified into four distinct functional gene categories: metabolism (transcription and regulatory functions), structural components (including head and tail proteins), Lysis genes, and hypothetical or other proteins, (Fig. 6, Supplementary Table S1).

### Metabolism

Twenty ORFs were annotated to play a role in the phage’s metabolism (Fig. 6 and Supplementary Table S1). Of the 20 ORFs, ORF2 was predicted as putative anti-sigma factor which is classified as an early gene plays a regulatory role in phage gene expression, especially by redirecting host RNA polymerase to recognize phage-specific promoters. ORFs 21 and 87 as putative endonuclease, which may regulate the phage replication including, host DNA Degradation and DNA recombination and Phage DNA packaging. ORFs 25 and 117 predicted as a DNA methylase usually has a role of serves to protect the phage DNA from degradation by the host's restriction-modification system. ORFs 26 and 82 as DNA helicase and DNA primase respectively, which plays a critical role in in the phage DNA replication and initiate DNA synthesis. ORF 83 for DNA polymerase which plays a key role as replication enzyme that synthesizes new copies of phage DNA. ORF 109 as tail terminator protein, which may play a crucial structural and assembly role in completing and stabilizing the phage tail structure. ORF 119 as terminate large subunit, which plays a central role in DNA packaging in the phage capsid.

### Structure

Eighteen ORFs were predicted to be involved in morphogenesis of phage PanM1EGY (Fig. 6 and Supplementary Table S1). Those includes ORFs 90, 91, 94 and 95 encoding tail fiber proteins, Tail proteins including ORFs 106, and 111. ORF 108 encoding genes for putative tail sheath protein, which typically involved in forming the contractile tail structure for Myoviridae. ORFs 96, 97, 99 to 102 for genes encoding phage baseplate proteins. Capsid (head) proteins including ORF 110 for putative phage minor head protein, and ORF 114 for putative major head protein, ORF 115 for putative decoration protein, ORF 118 as portal protein which plays an essential role in the assembly of the phage head) and in the DNA packaging and ejection processes.

### Lysis genes

Four ORFs (were predicted to be involved in lysis of bacterial cells (Fig. 6 and Supplementary Table S1). Those are 53 and 85 encoding a membrane protein which usually helps in target the bacterial membrane and contribute to infection, phage release, or interference with host physiology. ORF 65 that was annotated to encode cell wall hydrolase protein which degrade the peptidoglycan layer of the bacterial cell wall leading rupture and cell lysis. ORF 126 which is involved in the final step of host cell lysis, specifically targeting the outer membrane of Gram-negative bacteria. completes the lysis process after the endolysin and holin have disrupted the peptidoglycan layer and inner membrane, respectively.

### Lytic activity of the phages

**Fig. 7:**
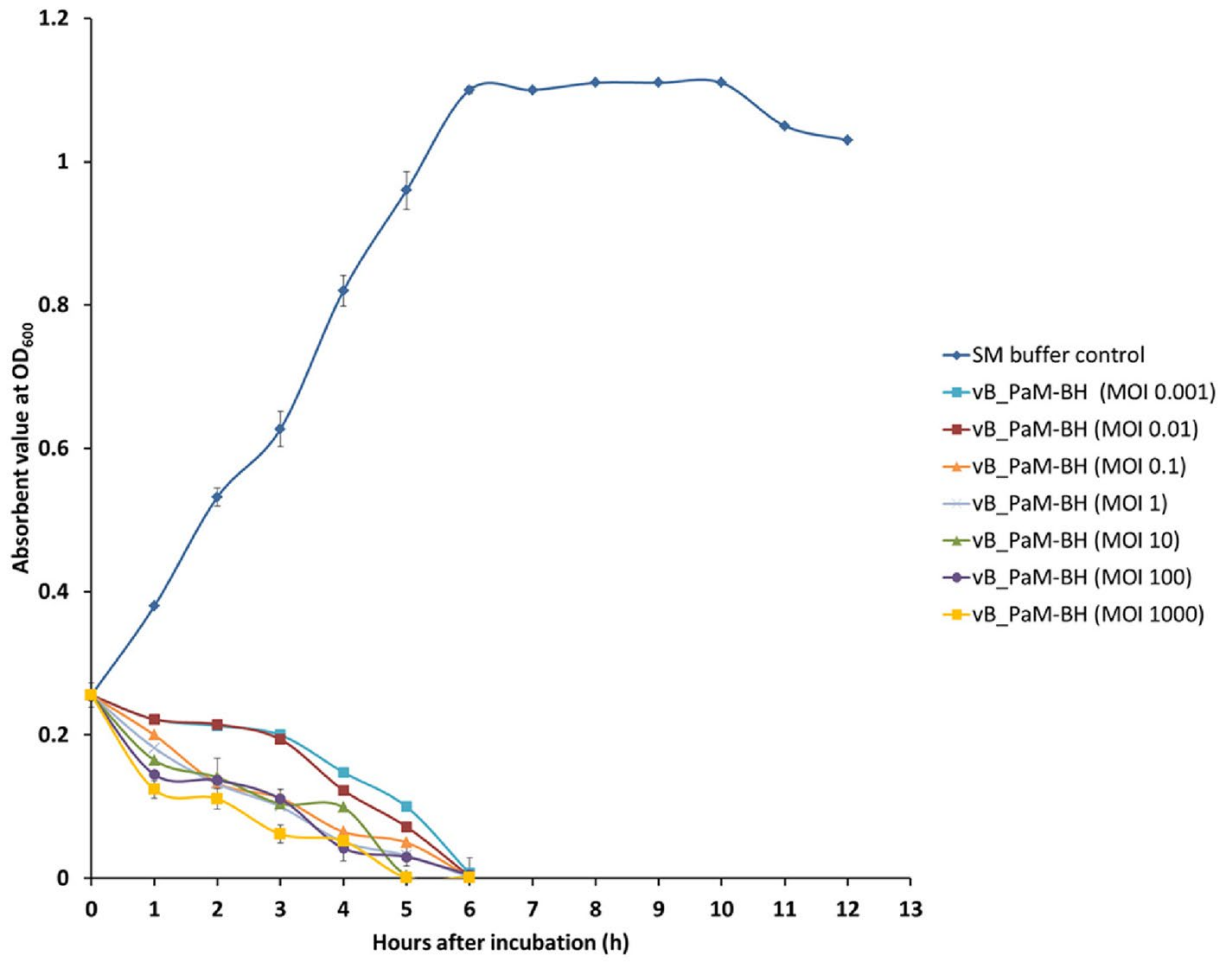
In vitro lytic activity of phage PanM1EGY. PanM1EGY was mixed with the King’s liquid cultures of the host bacterium *P. ananatis,* at MOIs of 0.001, 0.01, 0.1, 1 10, 100, and 1000. The optical density at 600 nm was measured every 1 h for a continuous period of 6 h

To assess the lytic efficacy of phage PanM1EGY, bacterial growth was monitored in King’s broth following treatment with various multiplicities of infection (MOIs) (Fig. 7). The phage exhibited a dose-dependent bactericidal effect, with higher MOIs leading to more rapid suppression of *P. ananatis* growth. Notably, at an MOI of 1000, significant inhibition was observed within the first 5 h. By the 6-h mark, phage treatments across all tested MOIs (0.001, 0.01, 0.1, 1, 10, and 100) showed clear antibacterial activity.

### Control of bacterial rot and leaf blight diseases caused by *P. ananatis* in potato, onion, garlic and strawberry using phage PanM1EGY

**Fig. 8:**
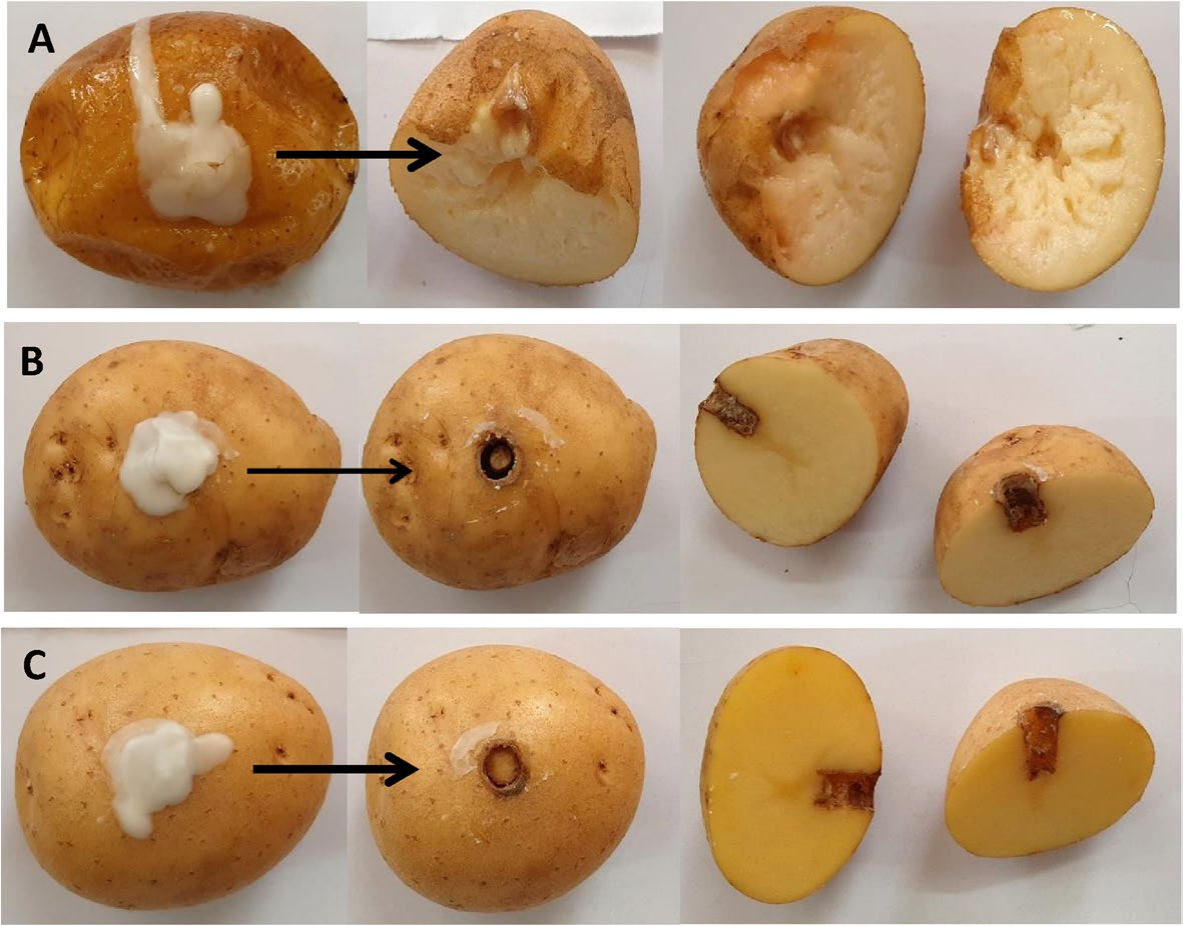
In planta assay demonstrating the protective potential of phage PanM1EGY *against Pantoea ananatis* in potato tubers. **A** Potato tubers inoculated with a bacterial suspension at 10⁹ CFU mL⁻^1^. **B** Potato tubers treated with sterile King’s broth serving as the negative control. **C** Potato tubers inoculated with bacterial suspension (10⁹ CFU mL⁻.^1^) in combination with phage PanM1EGY at a multiplicity of infection (MOI) of 1000

The biological control assays demonstrated that all positive control groups developed typical symptoms of aggressive rot disease on potato tubers. *P. ananatis* exhibited strong pathogenicity, causing extensive soft rot across the tuber tissues (Fig. 8). In contrast, no signs of rot were observed in tubers treated with phage PanM1EGY at an MOI of 1000, even after two weeks post-inoculation, similar to the negative control.

**Fig. 9:**
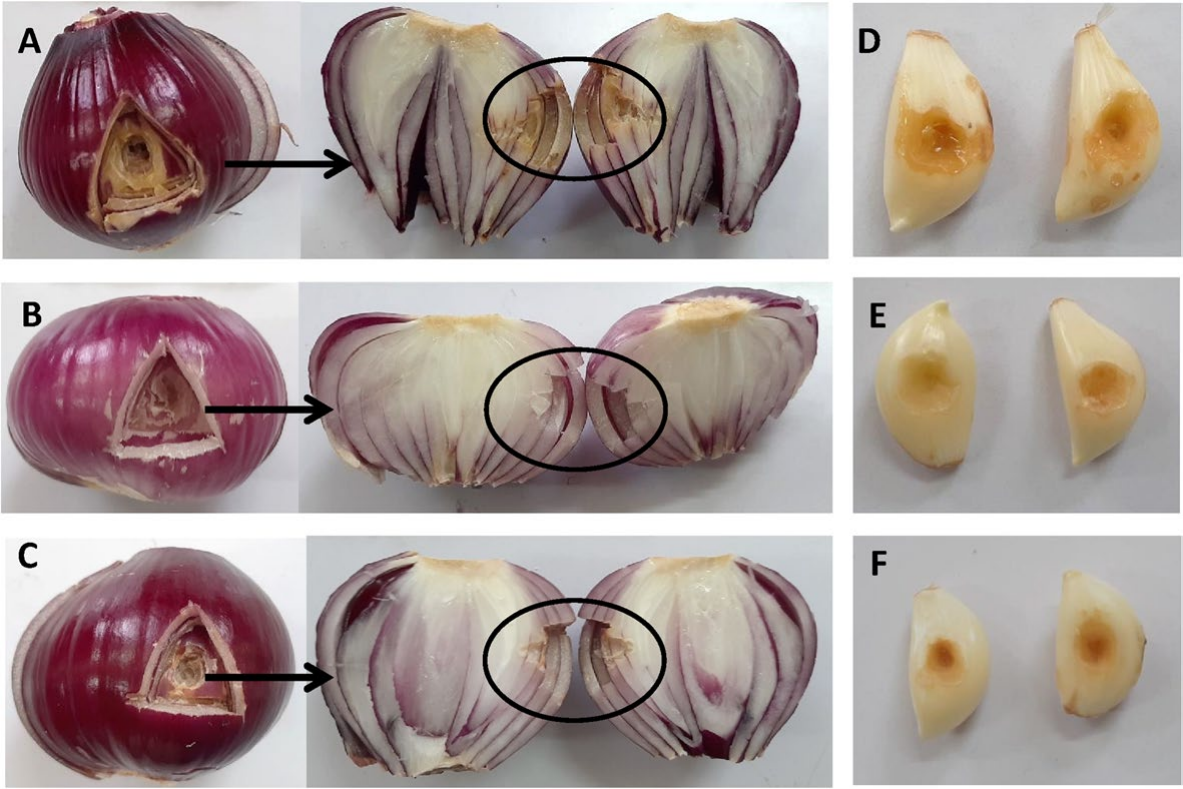
In planta bioassay illustrating the protective efficacy of phage PanM1EGY against *Pantoea ananatis* infection in onion bulbs and garlic cloves. **A** Onion bulbs inoculated with bacterial suspension at a concentration of 10⁹ CFU mL⁻^1^. **B** Onion bulbs treated with sterile King’s broth, serving as the negative control. **C** Onion bulbs inoculated with bacterial suspension (10⁹ CFU mL⁻^1^) in combination with phage PanM1EGY at a multiplicity of infection of 1000. **D** Garlic cloves inoculated with bacterial suspension at a concentration of 10⁹ CFU mL⁻^1^. **E** Garlic cloves treated with sterile King’s broth as negative control. **F** Garlic cloves inoculated with bacterial suspension (10⁹ CFU mL⁻.^1^) together with phage PanM1EGY at an MOI of 1000

In onion assays, *P. ananatis* caused rot symptoms, whereas phage-treated onions showed no signs of infection, consistent with the negative control (Fig. 9). Likewise, garlic cloves inoculated with *P. ananatis* developed severe rot symptoms; while those treated with phage PanM1EGY remained symptom-free, mirroring the negative control outcomes (Fig. 9).

**Fig. 10:**
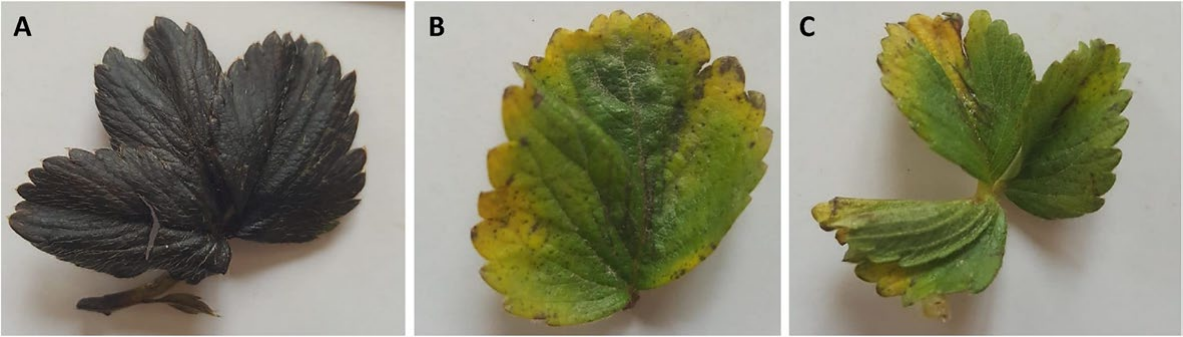
In planta bioassay for the protective activity of phage PanM1EGY against *P. ananatis* in strawberry. **A** leaf of strawberry was inoculated with bacterial cells at a concentration of 109 CFU mL − 1. **B** leaf of strawberry was inoculated with sterile King’s broth (negative control), and (**C**) leaf of strawberry was inoculated with bacterial cells 109 CFU mL − 1 and phage PanM1EGY at a multiplicity of infection (MOI) of 1000

Results of biocontrol of *P. ananatis* diseases in strawberry using PanM1EGY revealed that all positive controls showed characteristic severe leaf blight disease symptoms in the detached strawberry leaf test (Fig. 10), while no disease symptoms were recorded throughout leaves treated with phage PanM1EGY (Fig. 10). No leaf blight symptoms were also recorded on the negative control leaves.

## Discussion

The successful isolation and characterization of bacteriophage PanM1EGY highlight its strong potential as a biocontrol agent against *Pantoea ananatis*, a pathogen increasingly associated with soft rot and leaf blight diseases in economically important crops. We reported for the first time the lytic activity, genomic features, and biocontrol efficacy of *P. ananatis* infecting phage isolated from surface agricultural drainage water. The phage demonstrated clear lytic activity, producing well-defined plaques and spot lysis, which is indicative of its virulent nature. Such traits are essential for phages intended for agricultural use, as lytic phages are capable of rapidly lysing their bacterial hosts without integrating into the host genome. Using the bacterium *Pantoea ananatis*, which causes rice leaf blight all over the world, as the host, a new phage called PanM1EGY was identified in Egypt. Moreover, a novel phage known as PA-1 was isolated in Malaysia using the *Pantoea ananatis* as the host [49].

Transmission electron microscopy (TEM) serves as an essential method for the morphological analysis of bacteriophages, enabling the visualization of their complex structures and subsequent classification. Morphological analysis using TEM placed PanM1EGY within the class Caudoviricetes, which is known for including effective lytic phages. Similar result has been previously reported by Singh et al. [49], who confirmed that by using TEM, The phage PA-1, specific to *P. ananatis,* belonged to the Caudoviricetes class*.* The phage vB_PagP-SK1 infecting *Pantoea agglomerans* is a member of the Teseptimavirus genus within the Podoviridae family of the order Caudovirales [50]. Besides, *Pantoea agglomerans* infecting phages (Nifs112 and Nufs112), two podophages that appeared to vary primarily in their short non-contractile tail appearances, were isolated from a dead unidentified Latvian grasshopper specimen. However, it was found that phage Nafs113 had an elongated head, a less common myovirus morphotype [27]. Recent revisions by the ICTV highlight that the classification of phages is now predominantly based on genomic analysis [51]. In our study, phage PanM1EGY was found to infect all *P. ananatis* strains only of 27 tested different bacterial strains. The narrow host range exhibited by PanM1EGY is beneficial for targeted biological control, minimizing disruption to beneficial or neutral members of the microbial community. Similar host specificity has been noted in other *P. ananatis* phages [52], and this feature supports the environmentally responsible application of phage therapy in crop production systems. The phage vB_PagP-SK1 that infects *Pantoea agglomerans* was evaluated against 94 strains of the bacterium, which represent 10 known species. 15 strains in all were found to be susceptible. One of three *Pantoea brenneri* strains and one of nine *Pantoea septica* strains tested were also susceptible outside of the *P. agglomerans* group. *Erwinia billingiae* was sensitive; however, *Erwinia amylovora* was shown to be resistant [50].

The optimal multiplicity of infection signifies the initial ratio of phage to host that produces the greatest lytic efficiency. A reduced optimal MOI suggests that a lesser initial phage potency is necessary to lyse the maximum quantity of host bacteria, or to attain the highest potency of progeny bacteriophages. This decrease in the required initial phage potency diminishes the future expenses associated with phage commercialization [37]. The optimal MOI for phage PanM1EGY was 0.1. Because shorter latent periods and greater burst sizes suggest enhanced lytic efficiency, phages' one-step growth curve is useful for demonstrating their lytic efficiency. The phage PanM1EGY had a burst size of about 250 ± 2 PFU/cell, accompanied by a short latent period of 30 min. This suggests that PanM1EGY possesses a short latent time, robust lytic capability, and greater lytic efficiency [37]. The replication behavior of PanM1EGY, including a short latent period and robust phage production, aligns with what has been described for other lytic phages used in agricultural settings. Though variations exist between phages, these traits contribute to rapid infection cycles and effective bacterial suppression, which is desirable in managing fast-developing plant diseases.

Environmental tolerance is a key factor for field application, and phage PanM1EGY demonstrated stability under a wide range of temperatures and pH conditions that reflect those typical of Egyptian agricultural environments. After being exposed to UV light for 20 min, the phage used for this study can maintain stability, are highly adaptive to salt concentration up to 20%, and temperatures between 5 and 50 °C. Furthermore, phage exhibited stability in the pH range of 5 to 11, demonstrating their capacity to withstand harsh conditions. These results point to the phages' possible applicability for field applications. While most phages can endure temperatures ranging from 25 to 50 °C and pH values between 4 and 9, some are highly resistant to these environmental factors [53]. Its tolerance to heat, salinity, and pH variation supports its utility in diverse field conditions, echoing findings from previous studies on similarly resilient phages [52]. However, its sensitivity to high temperatures and UV exposure may necessitate protective formulations or strategic application timing to maintain efficacy in the field. Given that the survival rate of examined phage decreased with increasing exposure, the results imply that phage was sensitive to UV light. After 20 min of UV exposure, the phage titer dropped by approximately 1.83 log units, reaching 12.34 log. Numerous studies have attempted to lessen the impacts of UV radiation by using pure substances such astaxanthin and Tween 80, as well as natural components like plant extracts, casein, kaolin, and polysorbate 80, which can enhance phage stability and uptake in UV-stressed plants [54, 55].

The tolerance of different phages to salt concentrations varies. It's crucial to choose phages with higher levels of resistance by nature or to employ formulations that increase their stability in the intended environment [56]. The findings indicate that the phage PanM1EGY tested exhibited sensitivity to NaCl concentration, as their survival rate decreased with increased salt concentration. After 20% of NaCl concentration, phage PanM1EGY titer decreased by around 2.00 log units, reaching 12.17 log units. Formulations frequently encapsulate phages in protective materials such as polymers or nanoparticles, or use chemicals that stabilize the phage structure, to increase phage stability in high salt concentrations. By protecting it from the hostile surroundings, these techniques seek to keep the phage infectious [57, 58].

The phage’s dose-dependent inhibition of *P. ananatis *in vitro further confirms its lytic efficiency. Even at lower phage concentrations, antibacterial activity was observed, suggesting that PanM1EGY maintains effectiveness across a range of application scenarios. Phage PanM1EGY effectively inhibited the growth of *P. ananatis* strain Badr HH1within 5 h. This is consistent with observations made for other plant-pathogen-targeting phages and is crucial when considering economic and logistical aspects of field-level biocontrol applications.

Since their discovery at the start of the twentieth century, phages have been investigated as possible agents for controlling bacterial plant diseases. As a result of today's widespread chemical use, resistant strains have emerged and control effectiveness has decreased. Unlike chemical biocides, phages are naturally occurring in the environment; therefore humans are exposed to them every day without experiencing any negative effects [24].

The results of in vivo assays on potato, onion, and garlic demonstrated that phage PanM1EGY application effectively prevented the development of rot symptoms caused by *P.* *ananatis*, with results closely resembling those of negative controls. These outcomes underscore the phage’s promise as a postharvest treatment or pre-harvest intervention. The ability to protect multiple hosts broadens the potential use of this phage beyond a single crop, which adds to its practical value.

In the detached strawberry leaf test, application of the phage PanM1EGY remarkably suppressed bacterial leaf blight of strawberry. While without phage treatment, leaves showed severe leaf blight symptoms (the leaves quickly turned black Fig. (9)), and our results are consistent with those recorded by Das et al. [59]. Similarly, the suppression of bacterial palea browning of rice caused by *Pantoea ananatis* by phages was demonstrated by Azegami [26]. He found that, when one of the isolated phages was sprayed on rice plants during their flowering stage, both with and without a nonpathogenic strain of *P. ananatis*. When the phage and nonpathogenic *P. ananatis* were applied together, the disease symptoms were reduced in the sun. Applying the light-labile phage alone proved surprisingly suppressive [60].

Collectively, the results from this study suggest that phage PanM1EGY has the necessary biological and ecological attributes for development into a practical biocontrol tool. Further work should include formulation optimization, phage resistance monitoring, and field trials to evaluate performance under real agricultural conditions. Integrating phage-based solutions into existing integrated pest management (IPM) frameworks may offer a sustainable and environmentally friendly approach to mitigating bacterial diseases in crop systems.

## Conclusion

A lytic phage PanM1EGY was isolated from surface agricultural drainage water in the Dakahlia Governorate, Egypt, displaying a strong specificity for *P. ananatis* strain Badr HH1. The comprehensive characterization of PanM1EGY highlights its potential as an effective biocontrol agent against *P. ananatis*.

The genomic characterization of phage PanM1EGY revealed a linear dsDNA genome of 77,516 bp with 131 predicted ORFs. Comparative sequence analysis indicated substantial similarity to several related bacteriophages, particularly those infecting *Pantoea*, *Klebsiella*, and *Pectobacterium* species. Functional annotation highlighted genes associated with metabolism, structural proteins, DNA packaging, and lysis, alongside numerous hypothetical proteins.

PanM1EGY specificity, efficient replication, environmental stability, and demonstrated efficacy in both in vitro and in vivo assays support its application in managing bacterial rot and leaf blight diseases in crops such as potatoes, onions, garlic, and strawberry. Further field trials and formulation studies are warranted to optimize its use in agricultural practices.

## Supplementary Information

Supplementary Material 1.

## Data Availability

All data generated or analyzed during this research are included within the manuscript. The corresponding author is open to providing additional data or materials upon reasonable request.

## Abbreviations

*P. ananatis*: Pantoea ananatis
*PanM1EGY*: Name of isolated and identified phage
PFU: Plaque forming unit
EOP: Efficiency of plating
TEM: Transmission electron microscopy
MOI: Multiplicity of infection
MOIs: Multiplicities of infections
OD: Optical density
ICTV: International Committee on Taxonomy of Viruses
ORFs: Open reading frames
OrthoANI: Orthologous Average Nucleotide Identity
